# Planning the Surgical Correction of Spinal Deformities: Toward the Identification of the Biomechanical Principles by Means of Numerical Simulation

**DOI:** 10.3389/fbioe.2015.00178

**Published:** 2015-11-03

**Authors:** Fabio Galbusera, Tito Bassani, Luigi La Barbera, Claudia Ottardi, Benedikt Schlager, Marco Brayda-Bruno, Tomaso Villa, Hans-Joachim Wilke

**Affiliations:** ^1^IRCCS Istituto Ortopedico Galeazzi, Milan, Italy; ^2^LaBS, Department of Chemistry, Materials and Chemical Engineering, Politecnico di Milano, Milan, Italy; ^3^Center of Musculoskeletal Research Ulm, Institute of Orthopedic Research and Biomechanics, Ulm University, Ulm, Germany

**Keywords:** scoliosis, spinal deformity, finite element, deformity correction, spine biomechanics, patient specific

## Abstract

In decades of technical developments after the first surgical corrections of spinal deformities, the set of devices, techniques, and tools available to the surgeons has widened dramatically. Nevertheless, the rate of complications due to mechanical failure of the fixation or the instrumentation remains rather high. Indeed, basic and clinical research about the principles of deformity correction and the optimal surgical strategies (i.e., the choice of the fusion length, the most appropriate instrumentation, and the degree of tolerable correction) did not progress as much as the implantable devices and the surgical techniques. In this work, a software approach for the biomechanical simulation of the correction of patient-specific spinal deformities aimed to the identification of its biomechanical principles is presented. The method is based on three-dimensional reconstructions of the spinal anatomy obtained from biplanar radiographic images. A user-friendly graphical user interface allows for the planning of the desired deformity correction and to simulate the implantation of pedicle screws. Robust meshing of the instrumented spine is provided by using consolidated computational geometry and meshing libraries. Based on a finite element simulation, the program is able to predict the loads and stresses acting in the instrumentation as well as those in the biological tissues. A simple test case (reduction of a low-grade spondylolisthesis at L3–L4) was simulated as a proof of concept, and showed plausible results. Despite the numerous limitations of this approach which will be addressed in future implementations, the preliminary outcome is promising and encourages a wide effort toward its refinement.

## Introduction

Spinal deformities are relatively frequent pathologies which may appear in different stages of the human life, from childhood and adolescence to maturity and old age. Deformities may occur in a single anatomical plane, e.g., hyperkyphosis (Zaina et al., 2009) and sagittal imbalance (Barrey et al., 2011), or may be fully three dimensional in nature, as in many cases of adolescent idiopathic scoliosis (Schlosser et al., 2014). Despite conservative treatment, such as physiotherapy or bracing, could be a valuable option to improve the quality of life and to stop the progression of the deformity (Zaina et al., 2009), corrective surgical treatment may be necessary in more severe cases.

In decades of technical developments after the first surgical corrections of spinal deformities (Harrington, 1962; Nachemson, 1971), the set of devices, techniques, and tools available to the surgeons has widened dramatically. Recent reports show cases in which severe scoliosis [e.g., Xie et al. (2012) and Cecchinato et al. (2015)], sagittal imbalance (Berjano and Aebi, 2015), or congenital deformities, such as hemivertebra (Zhuang et al., 2015), have been surgically treated with advanced techniques and success rates, which would have been inconceivable 10 or 20 years ago. Nevertheless, the rate of complications due to mechanical failure of the fixation or the instrumentation remains rather high, especially regarding the most severe cases [e.g., Bianco et al. (2014)]. Indeed, basic and clinical research about the principles of the deformity correction and the optimal surgical strategies (i.e., the choice of the fusion length, the most appropriate instrumentation, and the degree of tolerable correction) did not progress as much as the implantable devices and the surgical techniques.

There is general consensus that the key problem in this regard concerns the anatomical and biomechanical inter-patient variability of the spinal deformity (Aubin et al., 2007), which appears to be so significant that even well-established classification systems have a relatively low usefulness for the choice of the optimal surgical strategy for a specific patient. In a rather recent paper, Aubin et al. (2007) submitted radiographic and clinical data about five patients suffering from adolescent idiopathic scoliosis to six experienced spine surgeons who were asked to provide their preferred surgical planning, and obtained a large variability in the responses, which was attributed to the lack of clearly defined strategies or rational rules. Indeed, spinal deformity surgery is still often regarded as an “art” in which the principles cannot be formulated in a clear way, and the personal experience of the surgeon still plays a vital role.

A precious support for the identification of the optimal correction strategies may come in the future from biomechanical simulations. The research group lead by Carl-Éric Aubin at the Ecole Polytechnique de Montréal devoted a vast amount of resources for the development of a Spine Surgery Simulator (S3), which could simulate based on a biomechanical model the outcome of surgical correction of spinal deformities, thus supporting the surgeon in the identification of the optimal strategy for a specific patient with scientifically solid predictions (Aubin et al., 2008; Majdouline et al., 2009). The simulator was designed to achieve a high degree of user-friendliness and speed, so that it could be proficiently used in a clinical setting. Despite its limited availability to potentially interested surgeons, the research project has proved its technical feasibility and clinical relevance by means of a strict validation process against documented surgical results (Aubin et al., 2008).

In this work, an alternative software approach for the biomechanical simulation of surgical correction of patient-specific spinal deformities is presented. The method basically differs from S3 in its aims, since it was designed to be a tool for the identification of the biomechanical principles of correction rather than in the ability to provide fast bedside predictions to be used for a specific patient in a clinical setting. To these aims, the more general framework of the finite element method instead of multibody dynamics simulation was chosen in order to potentially provide better insight into detail aspects, such as local stresses and strains in bone, intervertebral disks, ligaments, and spinal instrumentation, at the cost of higher computational requirements.

## Materials and Methods

### Implementation and Software Tools

Similarly to a previous study (Vergari et al., 2015), the proposed method is based on three-dimensional (3D) reconstructions of the spinal anatomy carried out with sterEOS software on biplanar radiographic images, which is described and validated elsewhere (Somoskeoy et al., 2012; Ilharreborde et al., 2014). The method is implemented in C++ and makes use of the QT development platform^1^ to manage the graphical user interface. 3D rendering and user interactions are based on the libQGLViewer library.^2^ All basic operations on triangulated surfaces are carried out by the free GNU Triangulated Surface (GTS) library.^3^ A number of more sophisticated processing steps, specified in the following paragraphs, are performed with Meshlab software^4^ through its scripting interface. Tetrahedral meshing is carried out by using the free Tetgen library (Si, 2011). Finite element models are solved with the commercial ABAQUS package (Dassault Systèmes Simulia Corp., Johnston, RI, USA) and automatically postprocessed by means of its Python scripting interface.

### Instrumented Spine Model

**Figure 1:**
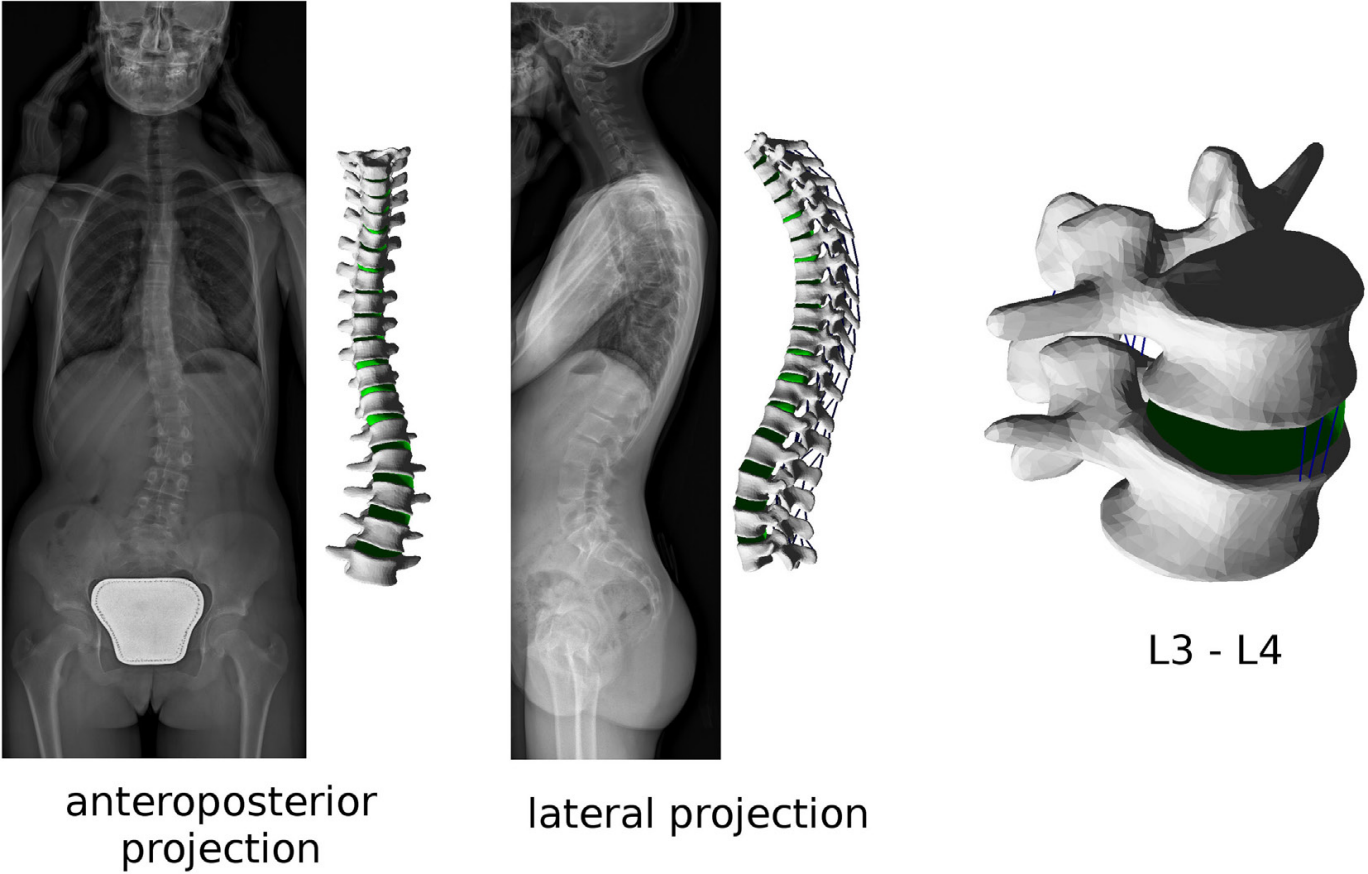
**Biplanar radiographic images obtained with the EOS Imaging System of a patient suffering from adolescent idiopathic scoliosis (left)**. Patient-specific finite element mesh of the thoracolumbar spine built with the present method, and (right) detailed view of the L3–L4 spinal segment of the same patient.

**Figure 2:**
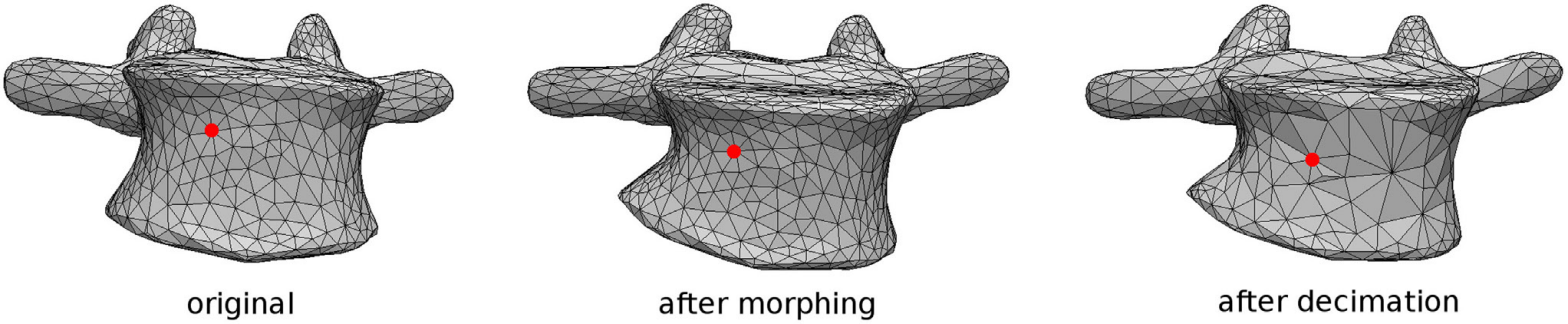
**Tracking of a specific node representing an anatomical landmark during the morphing procedure performed by sterEOS software, in which the surface mesh connectivity is not altered**. The closest node is then identified after coarsening of the surface (right).

As mentioned above, the patient-specific finite element model of the instrumented spine is built based on the 3D reconstruction of biplanar radiographic images (Figure 1). Triangulated surfaces of all vertebrae and pelvis in their correct anatomical position and orientation are directly extracted from the DICOM file including the reconstruction generated by sterEOS. This piece of software uses algorithm-based parametric models of vertebrae and an inference method (Humbert et al., 2009) to generate the 3D reconstruction of the spine, which always starts from the same triangulated surfaces for each vertebra. Taking advantage of this choice, the localization of specific landmarks and areas can be easily performed by identifying specific nodes in the deformed triangulated surface, the numbering of which was determined in the original, undeformed surface by visual inspection (Figure 2). Since the node positions are morphed together with the vertebra, this method allows for an accurate and repeatable identification of landmark points and vertebral regions, such as endplates, insertions of ligaments and facet joints.

**Figure 3:**
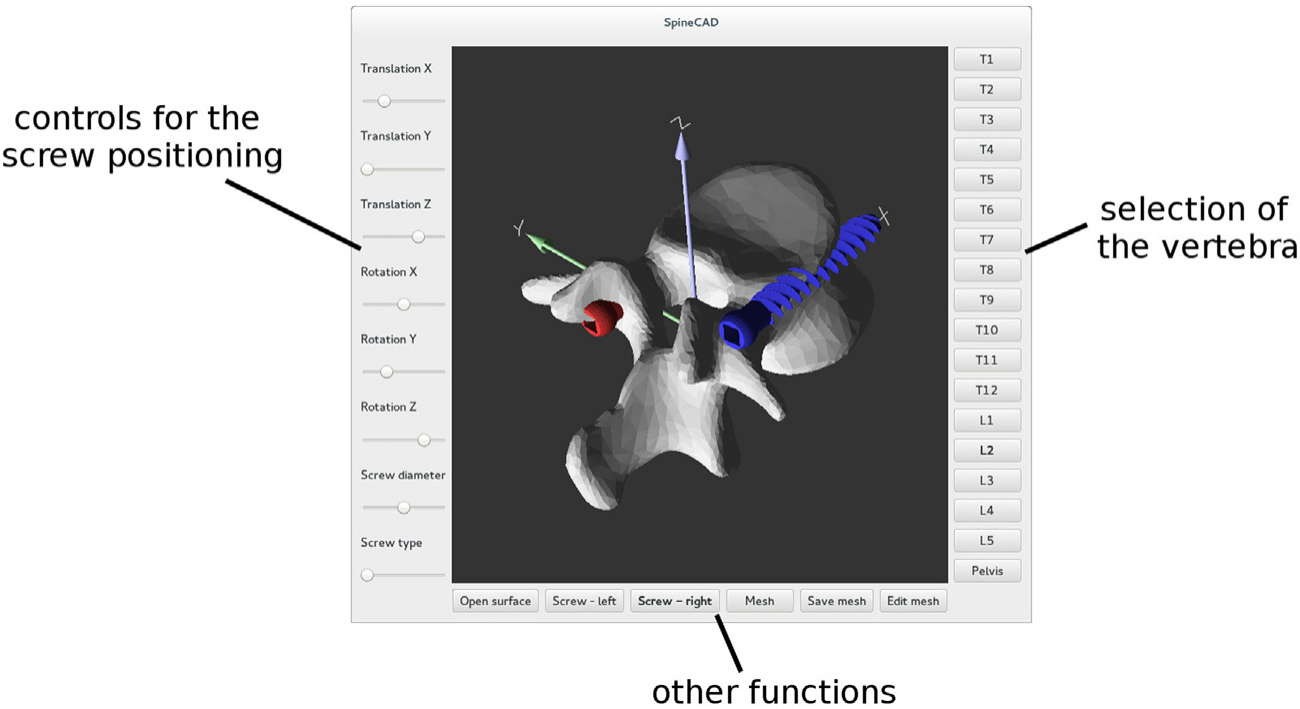
**Graphical user interface employed to properly position pedicle screws in each reconstructed vertebra**.

Pedicle screws are then positioned and oriented in the desired locations by using a graphical user interface (Figure 3). When the screw positioning is deemed optimal by the user, a new triangulated surface of the instrumented vertebra is generated by Boolean subtraction between the original vertebral surface and the surface of the screws. Tetrahedral finite element meshes of the vertebra and screws are then automatically generated. Prior to the generation of the 3D mesh, the user has the possibility to refine or coarsen the triangulated surface, in order to control the size of the final model.

**Figure 4:**
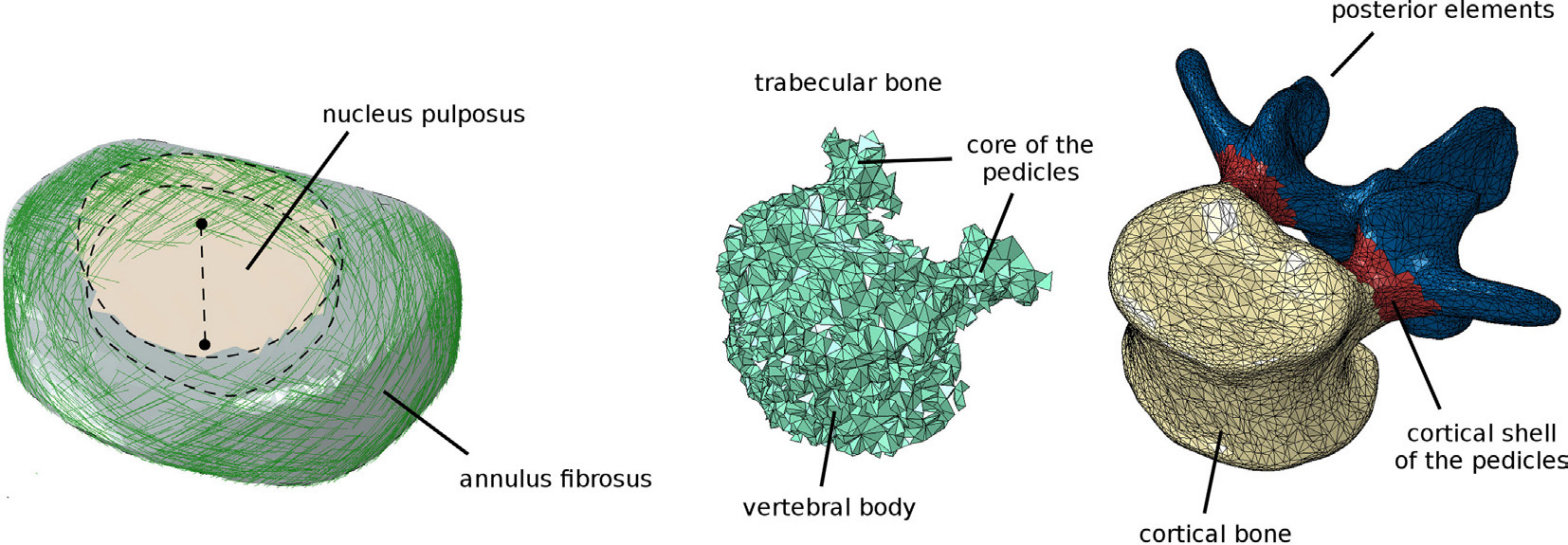
**Finite element model of the intervertebral disk (left), showing the cylindrical nucleus pulposus and the collagen fibers in the annulus fibrosus**. Assignment of the elements of the vertebra to distinct materials representing bone with different material properties (trabecular, cortical shell of the vertebral body and of the pedicles, posterior elements) (right).

When the volume meshes of two adjacent vertebrae have been generated, the intervertebral disk is automatically created. First, the surface nodes belonging to the endplate regions of the vertebrae are identified as described above. Then, the convex hull of the selected nodes is computed and resampled by means of the Poisson surface reconstruction algorithm (Hou et al., 2015) implemented in Meshlab. The elements belonging to a cylindrical region around the craniocaudal axis, the diameter of which is automatically calculated so that its volume is the 50% of that of the whole disk (Iatridis et al., 1996), are then identified as the nucleus pulposus (Figure 4), whereas the remaining elements constitute the annulus fibrosus. Annulus fibers are then created as non-linear springs arranged in order to have an average slope of ±30° with respect to the axial plane, by using a method described in detail elsewhere (Galbusera et al., 2006).

Six ligament groups are modeled as non-linear springs: anterior longitudinal ligament, posterior longitudinal ligament, flaval ligament, interspinous ligament, supraspinous ligament, and capsular ligaments. Most ligaments were modeled by means of three spring elements, except for the capsular ligament which was modeled with 12 elements following the border of the facet capsule and the supraspinous ligament, which was represented by a single spring. The contact between the facet joints was modeled with GAPUNI elements (Vena et al., 2005), with a fixed initial clearance of 0.4 mm and no friction (Schmidt et al., 2012). Nodes representing the ligament insertions and facet joints were located by using the method described above and represented in Figure 2.

All bony tissues were modeled as linear isotropic continua (Figure 4). Similarly as done for endplates, ligaments, and facet joints, nodes on the vertebral surface belonging to distinct zones (vertebral bodies, pedicles and posterior elements) were identified. Appropriate materials were then associated to the volume elements of the vertebra based on a proximity criterion, i.e., each element was associated to the surface node closest to the element centroid, and thus to the corresponding material. For the vertebral bodies and pedicles, elements were classified as cortical if belonging to the outermost layer, or trabecular elsewhere. A single material was used to represent laminae and spinous, articular, and transverse processes.

In this first implementation, the same material properties taken from the literature were used in the whole model and for all patients (Table 1). A preliminary validation was conducted in order to assess the plausibility of the spinal flexibility obtained with a model based on EOS data of a subject without any spinal pathology. No attempts were done yet for a proper, more comprehensive validation of the modeling approach or for a patient-specific calibration of the material properties.

**Table 1:** **Material properties used in the finite element models**.

		Elastic modulus (MPa)	Poisson’s ratio	Reference
	Cortical bone	12,000	0.3	Cowin (1991)
	Trabecular bone	200	0.315	Lu et al. (1996)
	Posterior elements	3500	0.25	Cowin (1991)
	Ligaments	Non-linear	–	Galbusera et al. (2011)
	Annulus: fibers	25	0.3	Galbusera et al. (2011)
Normal disks	Annulus: ground substance	4.2	0.45	Pitzen et al. (2001)
	Nucleus pulposus	1	0.499	Pitzen et al. (2001)
	Annulus: fibers	12.5	0.3	–
Unstable disk	Annulus: ground substance	2.1	0.45	–
	Nucleus pulposus	1	0.499	–

*“Unstable disk” summarizes the material properties used in the test case at the level of the spondylolisthesis in the models C–S and P–S*.

### Planning of the Deformity Correction Surgery

**Figure 5:**
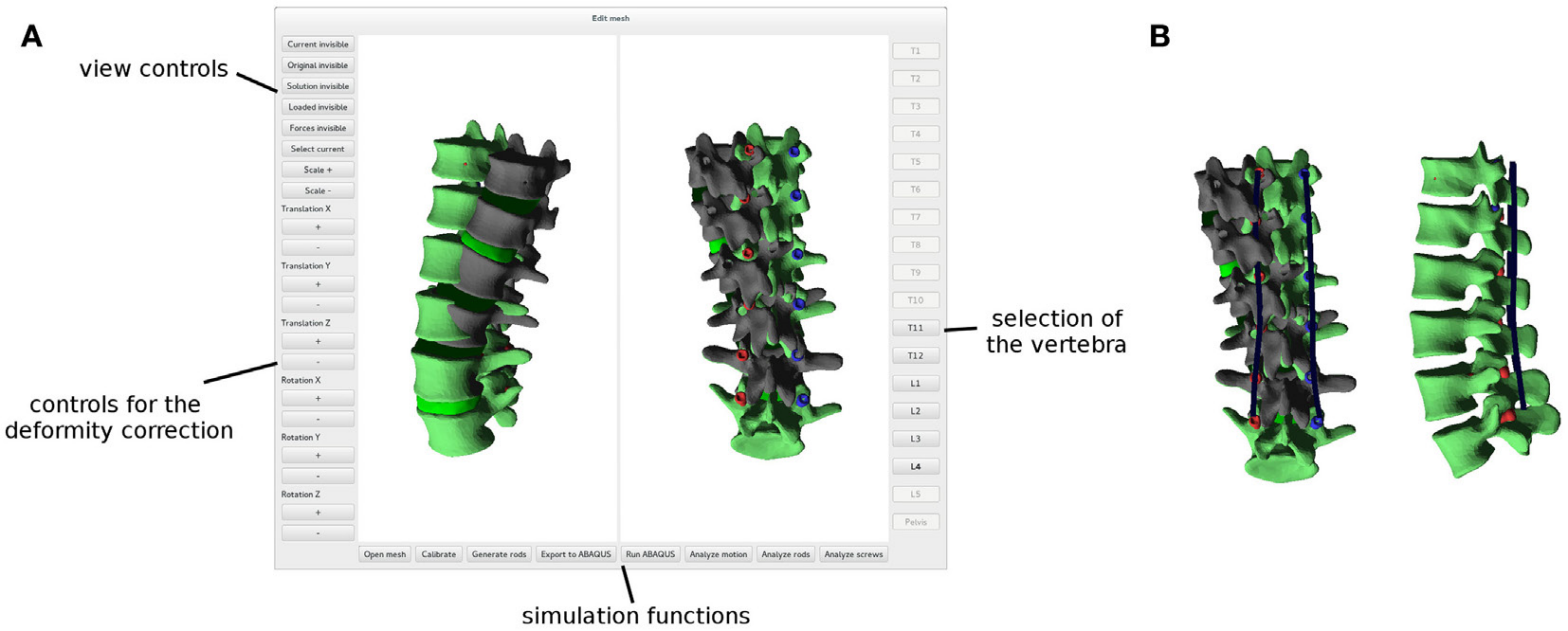
**Graphical user interface employed to plan the deformity correction (A)**. The original, deformed spine is shown in dark gray, whereas the planned correction in green. Posterior rods (in blue) automatically designed in order to optimally fit the desired shape of the spine **(B)**.

After a finite element mesh of the spine instrumented with pedicle screws has been generated, the desired surgical correction of the deformity is planned manually with a dedicated graphical user interface (Figure 5A). The user has the possibility to translate and/or rotate a single vertebra or a group of vertebrae by using buttons and sliders, until a satisfying desired correction has been achieved. The task is facilitated by a multiwindow 3D viewer and the possibility to show or hide the original finite element mesh in the deformed configuration.

Posterior rods are created when the planning of the correction is concluded. The contour of the rods is automatically generated based on the corrected spinal configuration as a bicubic spline passing through the screw heads, which are assumed in this implementation to move rigidly with the screw shaft, thus modeling a monoaxial screw (Figure 5B). The splines are then meshed with linear beam elements with material and geometrical properties mimicking those of the posterior rods of interest.

The user has the possibility to select, among all the pedicle screws present in the model, which screws should be connected to the left and right posterior rods, on the two sides independently. Despite allowing for unrealistic strategies, i.e., in the surgical practice, all pedicle screws are connected to the rods, this choice allows for an easy and fast comparison of different correction strategies by using the same finite element mesh.

Furthermore, the user has the possibility to simulate discectomies at selected levels, as commonly done in scoliosis surgery to facilitate the correction of the curves (Waisman and Saute, 1997). In this case, the intervertebral disk is completely removed at the chosen levels, as well as the anterior and posterior longitudinal ligaments. No kinematic or contact interactions were defined between adjacent vertebral endplates.

**Figure 6:**
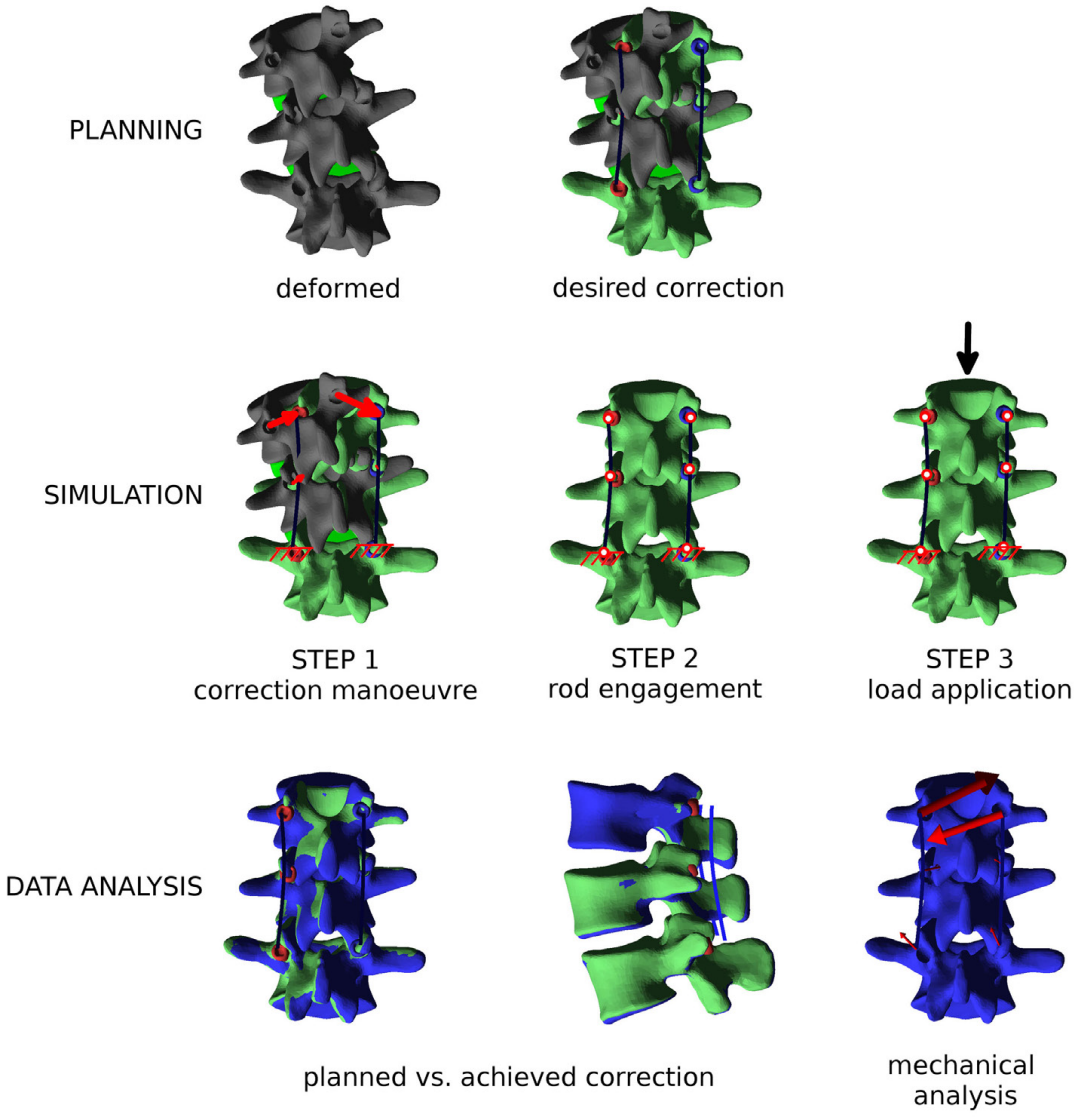
**Overview of the workflow to perform a biomechanical investigation of a spine deformity correction**. First (“PLANNING”), the three-dimensional correction is planned as desired with the graphical user interface, and posterior rods with appropriate contours are generated. Then (“SIMULATION”), the finite element simulation is performed in three steps: (i) the correction maneuver; (ii) rods and pedicle screws are locked together; and (iii) external loads are applied, if desired. Finally (“DATA ANALYSIS”), relevant mechanical outputs are extracted from the simulation and analyzed.

The simulation of the correction maneuver is then performed, in three loading steps as described below (Figure 6): (i) in the first step, displacement boundary conditions are applied to the nodes on the screw heads to reach the corresponding positions on the posterior rods, thus implementing the correction. The spine is, therefore, strained, and reaction forces are acting on the screw heads. The rods are fixed in space and not loaded, since the engagement between screws and rods is not modeled in this first step. The lower extremities of the posterior rods are constrained in all degrees of freedom. (ii) Kinematic couplings are created between the screw heads and the corresponding nodes on the rods, by means of a Fortran user subroutine, MPC (ABAQUS 6.10 Documentation, 2010). Boundary conditions on the screw heads are deactivated. The strain energy of the spine is released to the rods, which become strained and loaded as well. Upper and lower extremities of the model are not constrained except for the relevant screw heads. (iii) In the third optional step, external loads (e.g., a compressive load or a flexion–extension moment) are applied as desired. Boundary conditions are not changed, and the kinematic couplings remain active.

### Postprocessing and Data Analysis

When the finite element simulation is concluded, the ABAQUS output file is automatically processed by a Python script, which extracts the relevant results. First, nodal displacements are obtained and used to deform the mesh in the 3D viewer (Figure 5). Global orientations of the vertebrae before and after correction are geometrically calculated based on the original and deformed coordinates of landmark points. A direct comparison between the planned deformity correction and those actually achieved is, therefore, also possible.

Mechanical parameters describing the loads acting on the spinal instrumentation are also extracted. The forces acting between pedicle screws and vertebrae are calculated and plotted as vectors in the 3D viewer for an easy interpretation. Stresses and internal actions in the rods are also derived along the whole rod contours. In future implementations of the method, other relevant output values which may emerge as significant could be easily extracted via Python scripting.

### Test Case

**Figure 7:**
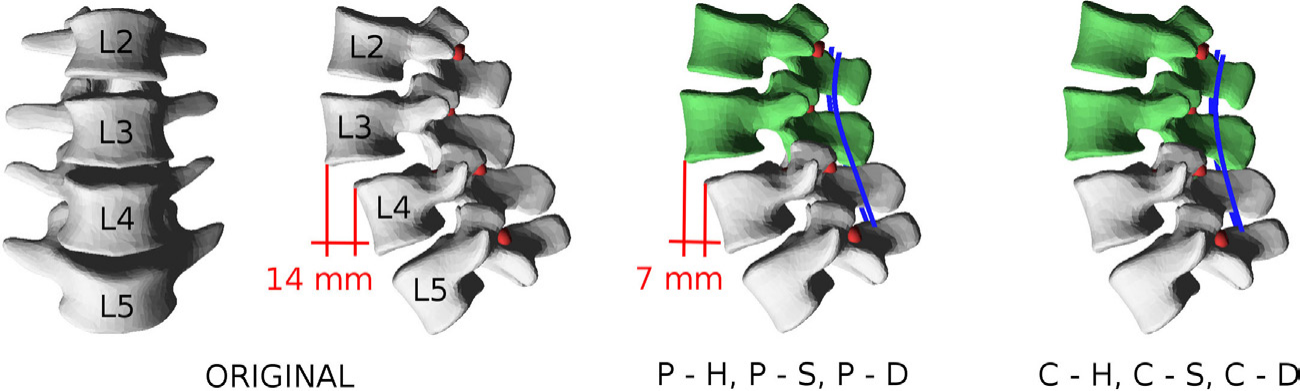
**Patient-specific model used as a test case**. In the pathological configuration (“ORIGINAL”), an anterior slippage of 14 mm is observable at L3–L4. Partial reduction to 7 mm is simulated by displacing L2 and L3 with the models “P–H,” “P–S,” and “P–D,” whereas complete correction is modeled with “C–H,” “C–S,” and “C–D.”

In order to test the plausibility of the results obtained with the approach, a simple case of spinal deformity has been selected as a benchmark. Biplanar radiographs of a patient suffering from grade I/II degenerative spondylolisthesis at L3–L4 (anterior slippage of 14 mm) have been collected. Apart from the listhesis, the patient had no other major spinal deformities. In the L2–L5 tract which was considered in the biomechanical model, minor coronal (6°) and axial (3°) asymmetries could be observed. Two possible surgical corrections were modeled: a partial reduction of the slippage of 7 mm and a complete reduction (Figure 7). In both cases, the correction maneuvers were purely translational, i.e., no rotational correction of the spinal shape was attempted. The spine was fixed in the corrected configuration by means of pedicle screws and posterior rods at L2–L5.

For each correction strategy, three cases were simulated: (i) no anterior surgery is performed, and the L3–L4 disk has the stiffness of that of a normal, healthy lumbar disk; (ii) no anterior surgery is performed, and the L3–L4 disk has half the stiffness of a normal disk (Table 1), thus modeling instability; and (iii) a complete discectomy is performed at L3–L4. Thus, six conditions were simulated: (i) partial reduction of the listhesis and healthy disk (“P–H”); (ii) partial reduction and “soft” disk (“P–S”); (iii) partial reduction and discectomy (“P–D”); (iv) complete reduction and healthy disk (“C–H”); (v) complete reduction and “soft” disk (“C–S”); and (vi) complete reduction and discectomy (“C–D”). For all models, the achieved correction was quantitatively compared to the desired correction in terms of vertebral orientations in the three anatomical planes. Furthermore, forces acting at the screw–bone interface as well as internal actions in the posterior rods were calculated.

## Results

### Usability of the Program

**Figure 8:**
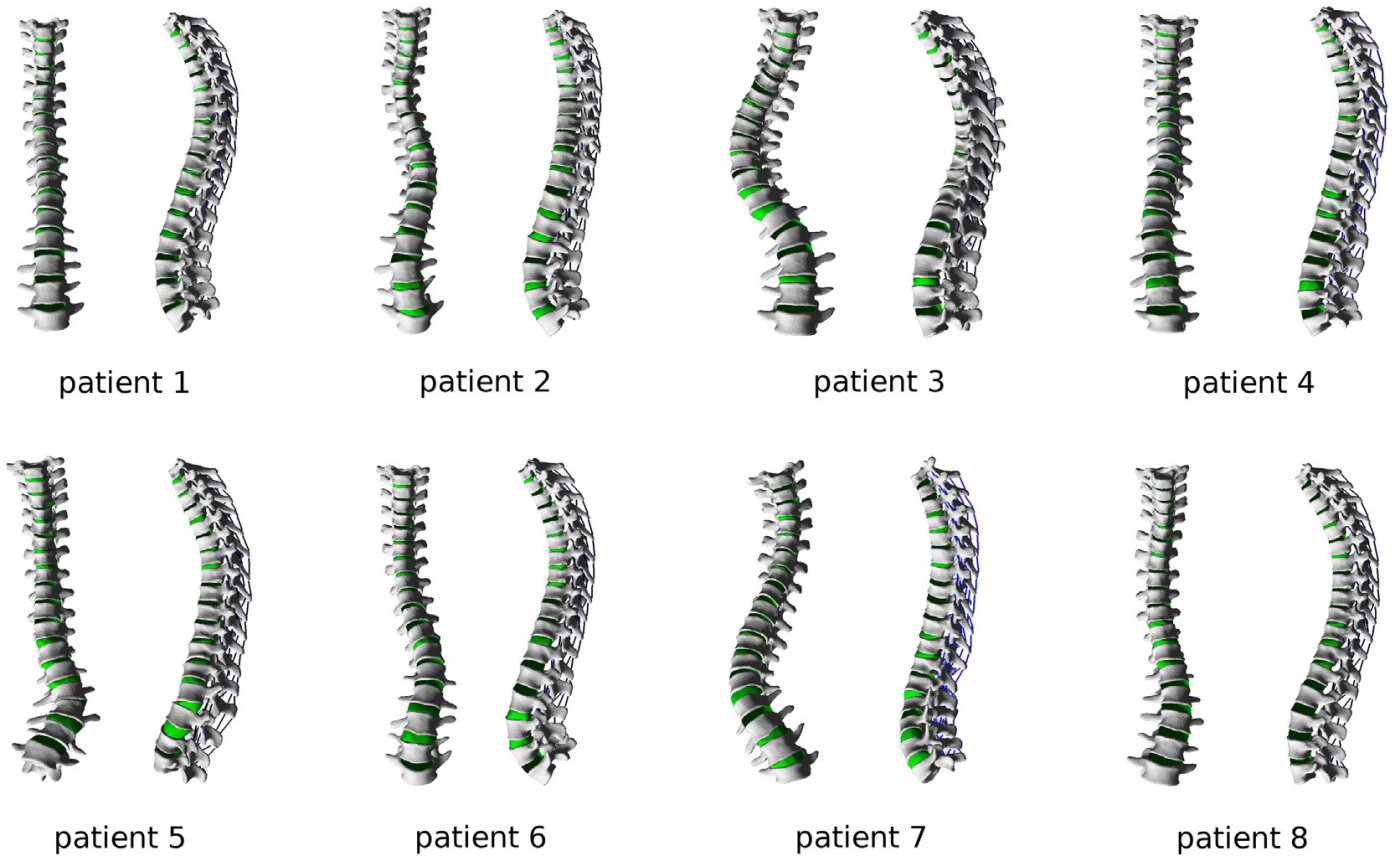
**Finite element meshes of the thoracolumbar spines of eight patients suffering from adolescent idiopathic scoliosis, ranging from mild to severe grades**.

To test the robustness and usability of the newly developed computer program, biplanar radiographic images of eight patients suffering from mild adolescent scoliosis were processed and finite element meshes of the whole thoracolumbar spines were generated (Figure 8). After 3D reconstruction of the images with sterEOS software, the computer program allowed for a robust meshing of all spinal anatomies, requiring ~15–30 min of work by a skilled operator for each case. The planning of the desired correction could also be consistently performed in 5–10 min. The ABAQUS simulation of the correction maneuver required a variable time dependent on the number of nodes in the mesh, and ranged between 15 min for short spinal segments with a coarse mesh to several hours for the whole thoracolumbar spines. As expectable, convergence issues emerged in some cases of extreme corrections involving high local strains, especially in the intervertebral disks. Automated postprocessing of the ABAQUS solution by means of Python scripting required <1 min.

### Test Case – Correction Potential of the Surgical Treatment

**Figure 9:**
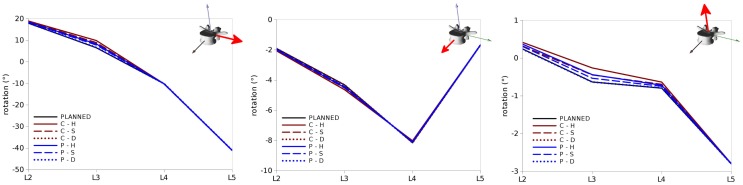
**Vertebral rotations predicted for the test cases in the sagittal (left), coronal (center) and axial (right) planes (in each panel around the axes highlighted in red) in the six configurations “C–H,” “C–S,” “C–D,” “P–H,” “P–S,” and “P–D”**. “PLANNED” represents the vertebral orientations of the desired correction.

All the various surgical strategies allowed for achieving a correction very similar to the planned one, with minor differences (Figure 9). In the sagittal plane, the maximal discrepancies were found for the orientation of L3, which ranged between 0° and 3° for the various configurations. As expectable, higher differences with respect to the planned alignment were found for the complete reduction scenarios, if compared to the partial reduction which required the application of a lower correction force. This effect was particularly pronounced for the C–H model, due to the higher stresses acting in the stiffer anterior structures.

Similar trends were observed for rotations in the coronal and axial planes (Figure 9), with maximal deviations found in both cases at L3. Nevertheless, the magnitudes of these differences were in all cases below 0.5° and could be, therefore, considered negligible.

### Loads on the Screws

**Figure 10:**
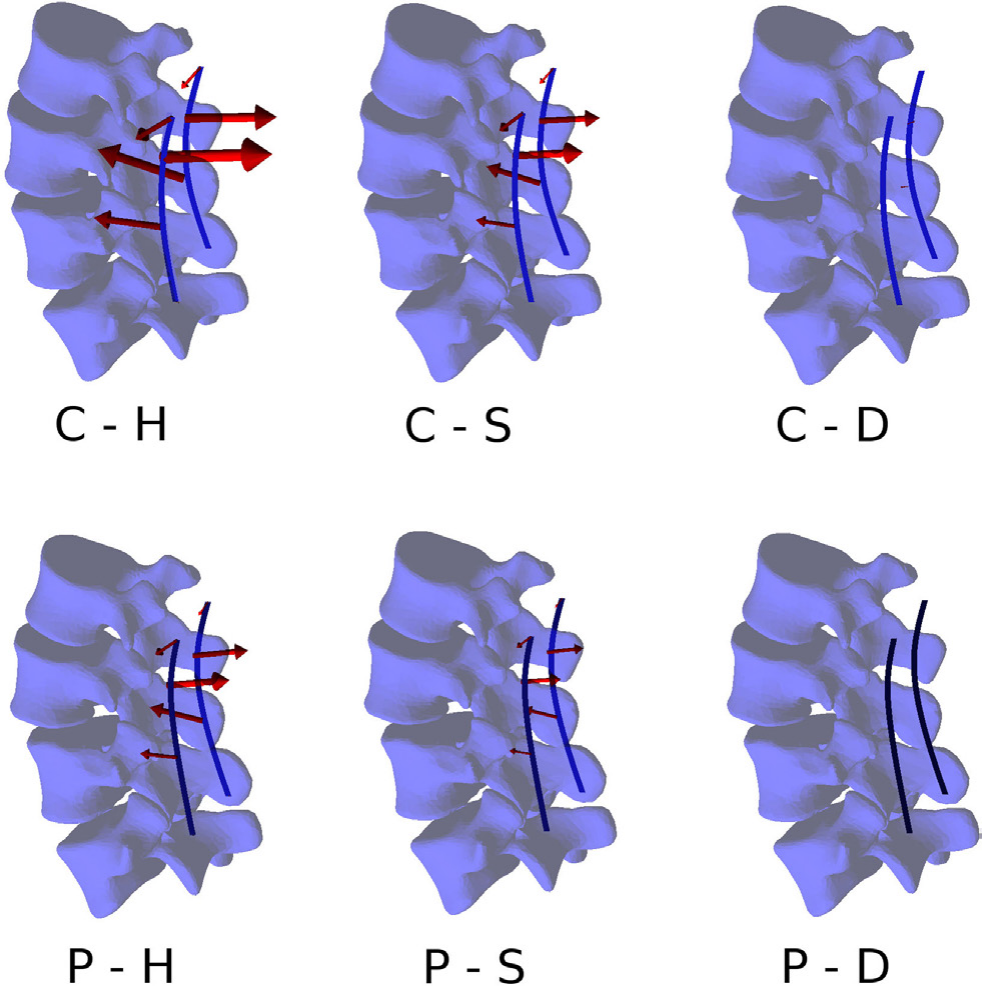
**Forces acting at the pedicle screw–bone interfaces, represented by the red arrows (sizes proportional to the magnitude of the forces), calculated for the six configurations**. Pull-out forces are predicted at L2 (arrows directed outwards), whereas push-in forces at L3 (arrows directed inward).

**Figure 11:**
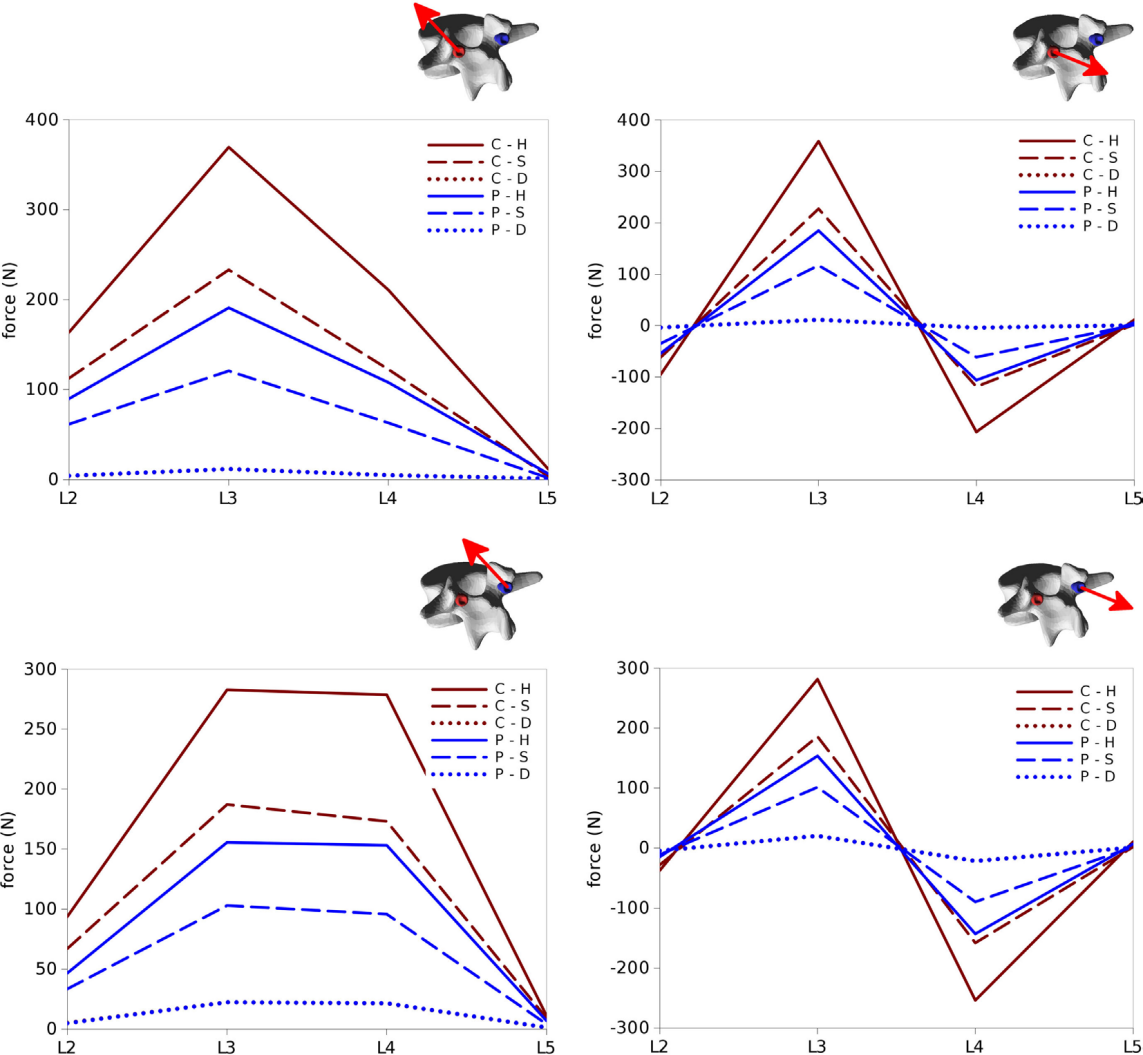
**Total forces (left) and pull-out forces (right) at the screw–bone interfaces for the screws on the left side (top) and those on the right side (bottom) predicted for the six configurations of the test case**.

Within the six configurations considered, the forces acting between each pedicle screw and the relevant vertebrae generally had the same directions and significantly differed only in magnitude (Figures 10 and 11). Coherently with the intuition, pull-out forces were found on the screws implanted in L3, whereas push-in forces with lower (on the left side of the instrumentation) or comparable magnitudes (on the right side) were predicted at the L4 level. Due to the proximity of the boundary conditions, negligible forces were calculated at L5, whereas the bone–screw interfaces at L2 were mainly loaded in the push-in, caudal direction.

The highest forces were predicted with the C–H, whereas C–S and P–H provided very similar values. The magnitude of the highest calculated forces (>300 N) may suggest a risk of screw pull-out in L3 for the C–H model (Zdeblick et al., 1993; Pfeiffer et al., 1996), whereas cut-out could be safely excluded due to the direction of the force being mostly aligned with the screw shaft. Complete discectomy at L3–L4 (C–D and P–D) allowed for a major reduction of the loads on the screws. Minor differences were found between the left and right side of the instrumentation; these deviations could be attributed to the slight asymmetries in the model, especially regarding the coronal orientation of L4 and the axial orientation of L2.

### Loads in the Rods

**Figure 12:**
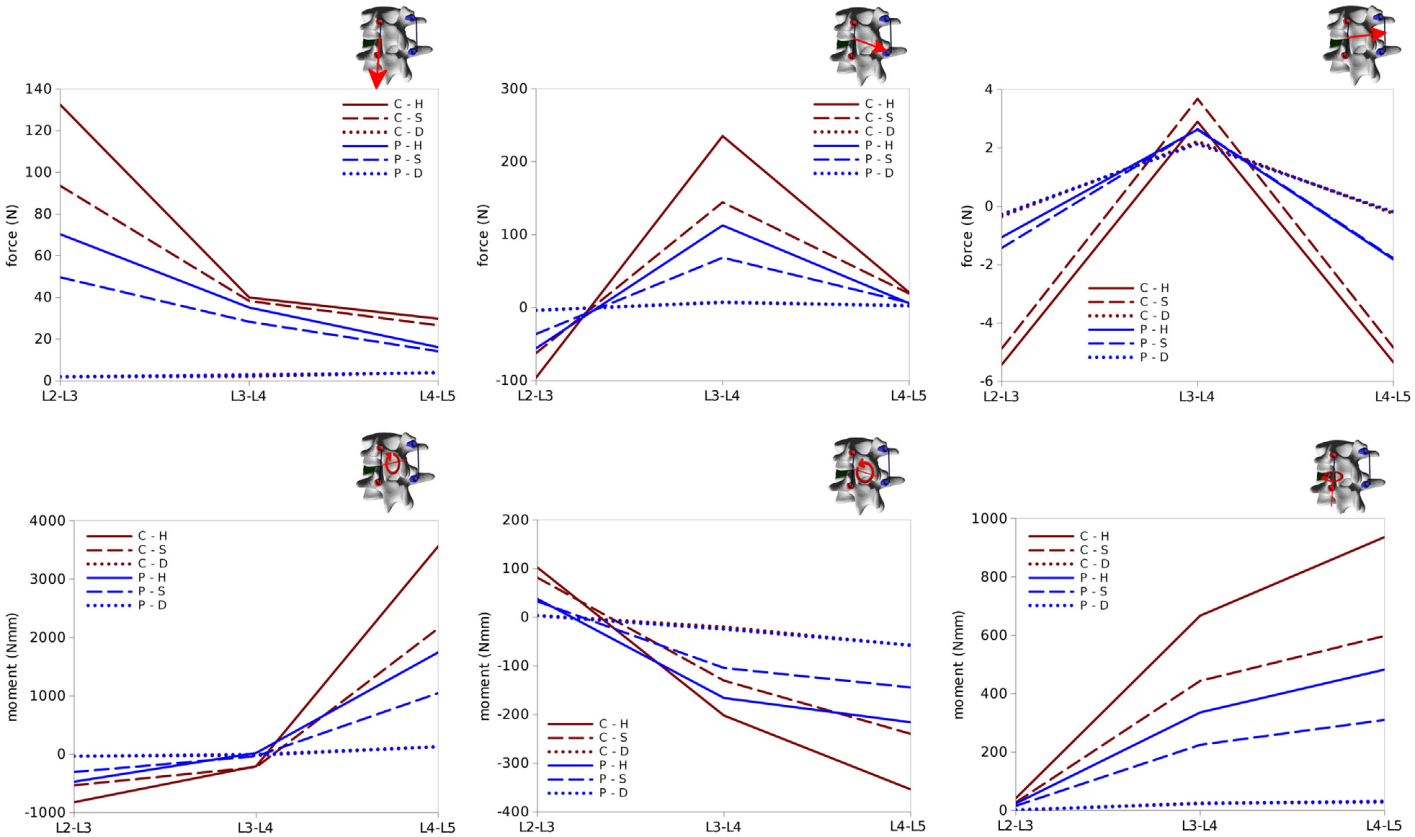
**Internal effects acting in the left rod of the test case**. First row, from left to right: axial force, anteroposterior shear force, laterolateral shear force. Second row: moment in the sagittal plane (flexion–extension), in the coronal plane (lateral bending), in the axial plane (torsion). Sign conventions are indicated by the right arrows.

**Figure 13:**
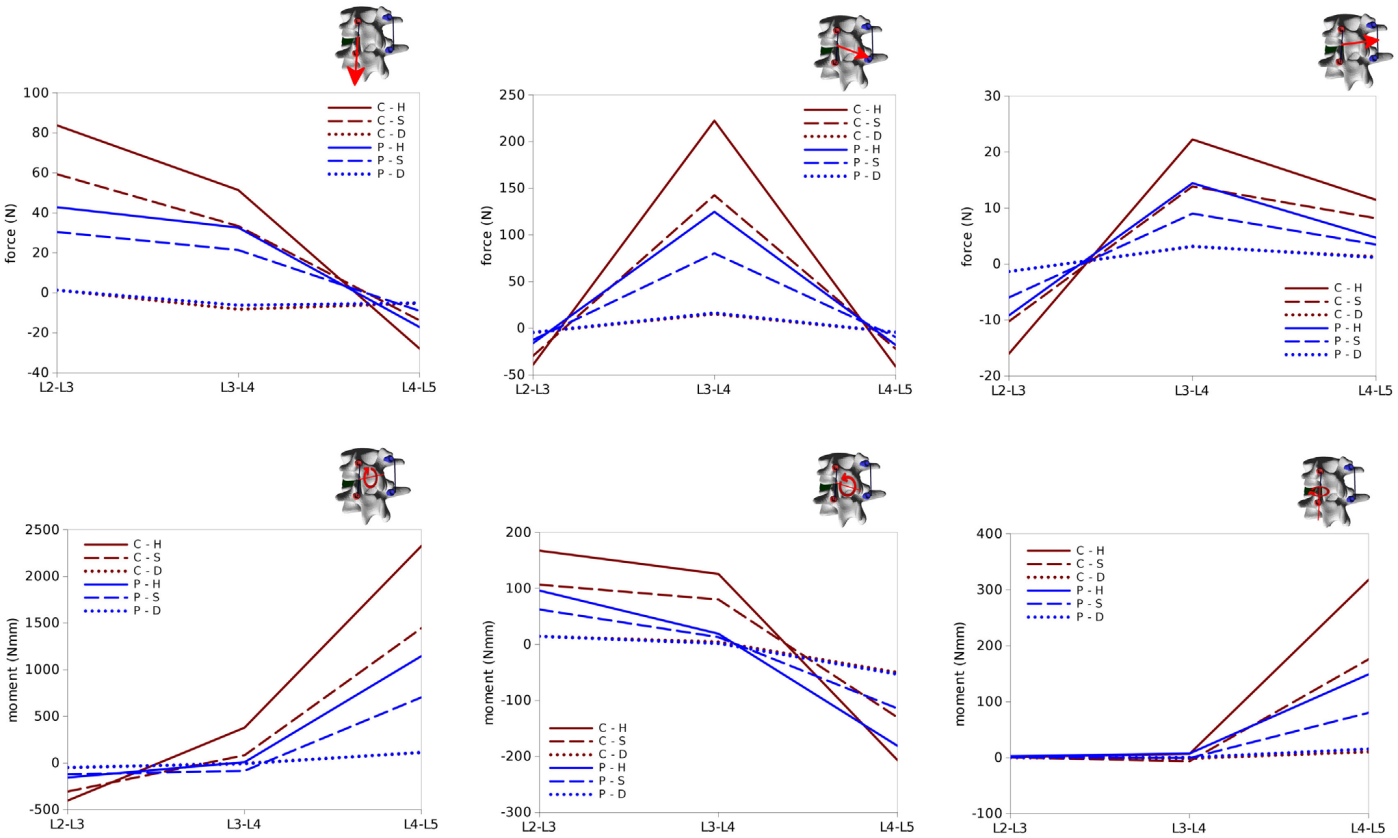
**Internal effects acting in the right rod of the test case**. First row, from left to right: axial force, anteroposterior shear force, laterolateral shear force. Second row: moment in the sagittal plane (flexion–extension), in the coronal plane (lateral bending), in the axial plane (torsion). Sign conventions are indicated by the right arrows.

Internal effects in the posterior rods also showed similar patterns at the left and right sides of the instrumentation, despite with some non-negligible differences, which may be attributed to the asymmetries in the spinal shape (Figures 12 and 13). Similarly on the two sides, anteroposterior shear forces had a significantly higher magnitude with respect to axial and laterolateral shear forces at the level of the spondylolisthesis reduction (L3–L4). On the contrary, axial forces where dominant at L2–L3, whereas laterolateral forces were low or negligible at all levels. Among the six configurations, patterns similar to those predicted for the screw forces emerged, with maximal load values for C–H, followed by C–S, P–H, and P–S, whereas negligible rod loads were calculated for C–D and P–D.

Bending and twisting moments in the rods showed higher differences between the two sides (Figures 12 and 13). Flexion–extension bending moments reached significant values at L4–L5, especially on the left side (+53% with respect to the right side), but were marginal at the level of the reduction as well as lower, and with opposite sign, at L2–L3. This finding is coherent with the intuitive expectations, for which the rods should be loaded mostly in anteroposterior shear at L3–L4 and in flexion–extension at the adjacent levels. Non-negligible values of the torsion moment were predicted at L4–L5, especially on the left side, and at L3–L4 on the left side only.

In summary, all the six configurations allow for a satisfactory correction of the spondylolisthesis, but differ significantly regarding the loads on the instrumentation and at the bone–screw interface. The only critical scenario appears to be C–H, which may induce a non-negligible risk of screw pull-out. In general, internal effects on the posterior instrumentation have magnitudes lower or comparable to those observed in physiological loading (Rohlmann et al., 1995), and therefore the rods should not be in danger of mechanical failure.

## Discussion

In this paper, a computational approach to simulate spinal deformity correction based on biplanar radiographic images of specific patients is presented. A user-friendly graphical interface allows the user to plan the desired correction strategy and to easily predict the achievable correction as well as mechanical variables, such as the stresses in the instrumentation and in the biological tissues. At this stage, the method is designed in order to be able to identify the biomechanical principles of deformity correction rather than as a bedside tool to be used to determine the optimal surgical strategy for a specific patient. In this respect, it differs from the previously mentioned S3 simulator, which is able to provide comparisons of various preoperative plannings including the various surgical maneuvers in a short time, being based on a relatively simple mechanical framework, such as multibody modeling. Nevertheless, the method also differentiates itself from other image-based programs, such as Surgimap (Akbar et al., 2013), which do not attempt to simulate the mechanics of the deformity correction but are limited to provide smart tools for measurements and comparisons.

**Figure 14:**
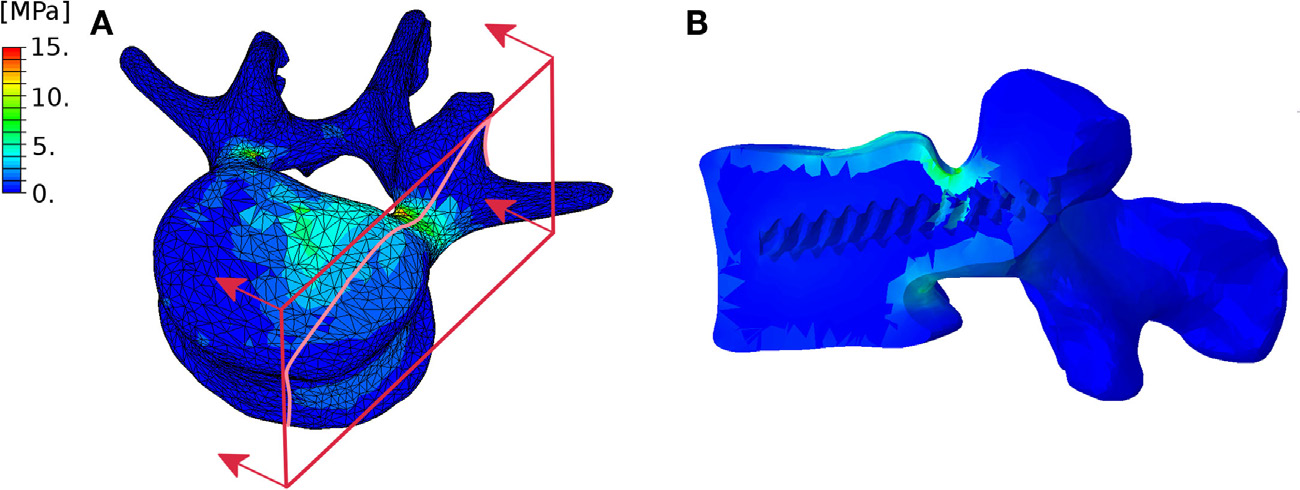
**Exemplary representation of the von Mises stresses in the L4 vertebra considered in the test case**. External **(A)** and section **(B)** views highlighting the higher stresses in the pedicles. The section plane used to create the section view is shown in red.

As a proof of concept, we selected a simple exemplary case of low-grade spondylolisthesis which allows for an intuitive analysis of the results, without attempting either to provide a detailed analysis of a complex case or to perform an extensive investigation of spondylolisthesis reduction. Nevertheless, the predictions were plausible and respected the general principles of deformity correction. Since there were only minor coronal and axial deformities, screw loads, and internal effects on the rods were nearly symmetrical with respect to the sagittal plane. Pull-out screw forces acted on the screws implanted in the vertebra which was displaced in the posterior direction, whereas push-in forces were predicted in the lower vertebrae. At the level of spondylolisthesis reduction, the major internal effect in the rods was the anteroposterior shear force, while in the adjacent segments flexion–extension moments were dominant. In addition to this general, qualitative understanding of the loads acting in the level subjected to correction and in the adjacent segments, the model provided a quantification of the effects, which may be useful to estimate the risk of hardware failure and loosening, which is currently left to the experience of the surgeon. It should be noted that the finite element model could potentially be used for a deeper analysis of the mechanical response, such as the quantification of local stresses (Figure 14) as well as sensitivity analyses, within the limitations of the modeling approach described below.

Only a few published papers proposed methods for the estimation of the instrumentation loads during and after deformity correction. In the studies conducted with the S3 simulator, topics, such as the optimum screw patterns to minimize the forces required to achieve the desired corrections in adolescent scoliotic subjects (Wang et al., 2012b), the efficacy of some correction maneuvers (Wang et al., 2011; Martino et al., 2013) and of different instrumentations (Wang et al., 2012a) were investigated. Abe and coworkers (Abe et al., 2015) predicted the corrective forces in 20 adolescent patients based on the changes in rod geometry and finite element analysis, based on post-operative CT scans. More frequently, the biomechanics of spinal deformity has been investigated by means of finite element models of representative cases (e.g., Rohlmann et al. (2006) and Agarwal et al. (2015)], thus not considering the strong intersubject variability typical of most spinal deformities.

Convergence issues emerged in some cases involving highly strained areas, especially in the intervertebral disks, and were arguably related to the local mesh quality. However, the approach used for the geometrical generation of the intervertebral disks provides a smooth, high quality surface mesh, which constitutes an appropriate input for volume meshing (Hou et al., 2015). Nevertheless, local mesh refinement may improve convergence in selected cases, and should be taken into account as a possible future development.

Despite the potential of our approach in exceeding the limitations of the published works, many aspects relevant to the biomechanics of spinal deformity correction were not covered in the present implementation. First and foremost, only monoaxial pedicle screws were modeled. Polyaxial screws as well as hooks are fundamental tools which are used by most surgeons, even in hybrid constructs, and therefore support for them is needed. As a matter of fact, the revolutionary Cotrel–Dubousset approach to treat idiopathic scoliosis, which is still the base for many current instrumentation systems, included both polyaxial screws and hooks (Cotrel and Dubousset, 1984). Other surgical techniques which are not currently supported are spinal osteotomies, such as Ponte and pedicle subtraction osteotomies, which are widely used for the treatment of adult sagittal imbalance (Gill et al., 2008), and other specific instrumentations which may be used in some cases, such as interbody cages, crosslinks, double-rod systems with dominos and sublaminar wires.

Other limitations pertain to the modeling approach itself. Despite comprehensive biomechanical data are not available yet and preliminary studies appeared only very recently (Mannen et al., 2015), it is known that the rib cage has a considerable stiffness and may further reduce the flexibility of the trunk (Oda et al., 2002), with considerable effects on the forces necessary to achieve the correction and the loads acting on the instrumentation. Rib humps which may be present in severe scoliotic cases may also have an influence on the correction procedure (Clin et al., 2006), but the extent and relevance of this effect is nowadays unknown and cannot, therefore, be simulated.

No boundary conditions or other mechanical constraints were imposed to the upper and lower extremities of the spine model, which were, thus, free to move without any resistance. This assumption collides with the real corrections in which other anatomical structures, such as head and pelvis, are connected to the spine and limit its ability to be deformed during the correction maneuvers. Another simplification concerns the connection between screws and rods by means of the MPC user subroutine neglecting significant information about the shape of the instrumentation, and thus preventing to use rod stresses obtained in proximity of the screws to evaluate the risk of hardware failure in this region due to possible numerical artifacts and low accuracy.

Figure 14 highlights the approximate nature of the proximity criterion used to assign the material properties to the bone tissue. As a matter of fact, this approach does not allow for the precise definition of a cortical shell with a predefined thickness, and may cause irregular stress distributions. An approach including the explicit definition of separate cortical and trabecular volumes is currently being developed.

The material properties of the biological tissues were taken from the literature and were mostly derived from experiments conducted on healthy specimens. As a matter of fact, patients with deformities may exhibit a stiffening of the spinal soft tissues, to an extent which is strongly variable from patient to patient and from level to level (Lafon et al., 2010). At the same time, many elderly patients suffer from osteopenia or osteoporosis, which were shown to have a non-negligible prevalence also in adolescent scoliotic subjects (Ishida et al., 2015) and significantly reduce the stiffness and strength of the bony tissues. Therefore, it appears that an effort toward the incorporation of patient-specific material data as they could be derived from biomedical imaging (Lafon et al., 2010) is required in order to improve the accuracy and reliability of the numerical predictions, and is planned as a future development. Another possible strategy to integrate deformity-dependent material properties is given by intra-operative measurements of the segmental stiffness by means of instrumented forceps (Klockner et al., 2003; Reutlinger et al., 2012) or stepper motor-based equipment (Brown et al., 2002), which may be correlated with the radiological appearance of the spine and thus exploited to implement localized material properties. For the sake of simplicity, bone was modeled as a simple linear elastic continuum; a more sophisticated formulation including a failure criterion would allow for more accurate predictions of the risk of loosening of the instrumentation as well as of vertebral fracture.

The main limitation of the present work is, however, the lack of model validation. As a matter of fact, patient-specific biomechanical models of deformed spines are nearly impossible to validate, due to their inherent variability and the lack of data apart from radiological imaging or clinical assessment. Experimental testing with cadaveric spines has been extensively used for the validation of numerical models, but is largely unpractical for the investigation of spinal deformities. As a matter of fact, deformed spine specimens are very rare and exhibit a strong inter-specimen variability, which prevents a proper repeatability and statistical analysis of the results. Alternative methods [e.g., deforming physiological specimens, such as in Wilke et al. (2015)] are currently under development, but have significant limitations and cannot be considered as consolidated methods.

A proper verification of the finite element models is, however, possible and necessary before the predictions could be used to extract clinically relevant information (Viceconti et al., 2005). In particular, mesh sensitivity studies are required in order to ensure that the predictions are mesh independent. It should be noted that the method allows for an arbitrary refining or coarsening of the meshes, by using algorithms available in the Meshlab and TetGen computer programs.

In summary, taking into account our aim of determining the biomechanical principles of deformity correction rather than accurately modeling a surgical procedure for a specific patient, we believe the preliminary outcome of our approach is very promising, and encourages a wide effort toward its refinement.

## Conflict of Interest Statement

The research was conducted in the absence of any commercial or financial relationships that could be construed as a potential conflict of interest.

## Footnotes

^1^http://www.qt.io

^2^http://libqglviewer.com/

^3^http://gts.sourceforge.net

^4^http://meshlab.sourceforge.net/
